# Forecasting Individual Patients’ Best Time for Surgery in Colon-Rectal Cancer by Tumor Regression during and after Neoadjuvant Radiochemotherapy

**DOI:** 10.3390/jpm13050851

**Published:** 2023-05-18

**Authors:** Emanuele Martorana, Paolo Castorina, Gianluca Ferini, Stefano Forte, Marzia Mare

**Affiliations:** 1 Istituto Oncologico del Mediterraneo, 95029 Viagrande, Italy; 2 Istituto Nazionale di Fisica Nucleare (INFN), Sezione di Catania, 95123 Catania, Italy; 3 Faculty of Mathematics and Physics, Charles University, V Holešovičkách 2, 18000 Prague, Czech Republic; 4 REM, 95029 Viagrande, Italy

**Keywords:** neoadjuvant radiotherapy, mathematical model, response prediction

## Abstract

The standard treatment of locally advanced rectal cancer is neoadjuvant chemoradiotherapy before surgery. For those patients experiencing a complete clinical response after the treatment, a watch-and-wait strategy with close monitoring may be practicable. In this respect, the identification of biomarkers of the response to therapy is extremely important. Many mathematical models have been developed or used to describe tumor growth, such as Gompertz’s Law and the Logistic Law. Here we show that the parameters of those macroscopic growth laws, obtained by fitting the tumor evolution during and immediately after therapy, are a useful tool for evaluating the best time for surgery in this type of cancer. A limited number of experimental observations of the tumor volume regression, during and after the neoadjuvant doses, permits a reliable evaluation of a specific patient response (partial or complete recovery) for a later time, and one can evaluate a modification of the scheduled treatment, following a watch-and-wait approach or an early or late surgery. Neoadjuvant chemoradiotherapy effects can be quantitatively described by applying Gompertz’s Law and the Logistic Law to estimate tumor growth by monitoring patients at regular intervals. We show a quantitative difference in macroscopic parameters between partial and complete response patients, reliable for estimating the treatment effects and best time for surgery.

## 1. Introduction

Colorectal cancer is one of the most frequently occurring types of malignant neoplasm, and it represents the second cause of cancer after prostate and breast cancer, respectively, in men and women [1]. Among all types of colorectal cancer, locally advanced rectal cancer (LARC) has an incidence of about one-third [2], and a precise staging requires at least 12 lymph nodes to be retrieved during surgery, according to the American Joint Committee on Cancer (AJCC). The last few decades have witnessed a radical change in outcomes in patients with LARC thanks to neoadjuvant therapy (NAT), which has also been demonstrated to reduce the lymph node yield (LNY) [3,4]. The standard treatment of LARC consists of preoperative radiotherapy and chemotherapy, used alone or in combination followed by surgery, with a total mesorectal excision (TME) technique [5] and postoperative adjuvant chemotherapy, if needed, with fluorouracil and oxaliplatin that confers excellent local control [6]. Even if TME is part of the standard of care for LARC, its impact on the quality of life (QoL) for patients that achieve a clinical complete response (cCR) is noticeable [7], preferring investigations of non-operative management (NOM) strategy, also known as the “watch-and-wait” approach. Since 2018, the National Comprehensive Cancer Network (NCCN) guidelines [8], thanks to the positive results of novel neoadjuvant therapy (NAT) in terms of tumor response, have included the option for Total Neoadjuvant Therapy (TNT) to achieve better outcomes and QoL [9]. In the case of TNT treatments with cCR and NOM, patients experience a strict follow-up schedule, assessing: clinical condition, lymph node status (with imaging), endoscopic examination, etc., chemoradiotherapy (NACRT); however, with the addition of ChT with 5-fluorouracil, capecitabine or oxaliplatin to radiotherapy (RT) before surgery, and reduced local recurrence (LR) with no effects on the Overall Survival (OS) [10,11,12,13,14]. Nowadays it is the standard therapy for locally advanced rectal cancer. Predictors of outcomes are currently taken into great consideration, especially regarding tumor regression grade (TRG) and ypStage, although these data are obtainable only as post-operative [15,16,17]. In this context, the oncological community is eager to identify biomarkers predictive of tumor response before treatment [18,19,20].

After neoadjuvant chemotherapy (NACT), between 10% to 25% of patients with LARC have a pathologic complete response [21] that increases for patients subjected to high-dose NACRT [22]. Again, tumor volume regression due to NACT can give useful information on the level of patient response. Indeed the volume reduction still persists many weeks after the end of therapy, and the best time for surgery is considered between 8–12 weeks [23] after the completion of the treatment. Recently, a study [24] based on 116 enrolled patients with middle and lower rectal cancers suggested that the time interval of 10–12 weeks can be considered optimal because comparable clinical and perioperative outcomes and preferable oncological outcomes were observed for intervals of this length. The results of ref. [25] suggest a prolonged time interval between the end of chemoradiation and oncological resection in patients with locally advanced rectal cancer can be beneficial for higher rates of pathological complete response as well as the tumor regression grade without increased perioperative morbidity.

The time to tumor response to radiation therapy (RT) changes among different histologies and depends on cancer cell radiosensitivity, oxygen landscape, vasculature, cell composition, etc. [26,27]. Moreover, tumor volume can increase or decline, according to some other factors like, for example, the interaction of the microbial community with the host, which modifies the immune response, metabolism, and oncogenesis [28]. Therefore, the microscopic dynamics, which produces the different evolution patterns of the tumor size, is a complex phenomenon where different cell subpopulations (different clones of cancer cells, tumor-associated fibroblasts, resident mesenchymal cells, infiltrating immune cells, etc.) are involved [29].

Mathematical models in radiation biology and physics arose after the first radiotherapy treatment about 100 years ago, and played a fundamental role in understanding the evolution of cancer under ionizing radiation. The most well-known model is the Linear-Quadratic (LQ) that describes cell survival after a single radiation dose [30]. A quantitative description of the tumor shrinkage during and after NACRT has been recently proposed [31] on the basis of general, macroscopic evolution patterns by using two tumor growth laws: Gompertz [32] and Logistic [33]. This model, based on a very small number of parameters such as the initial exponential growth and the carrying capacity [34,35], clearly selects between responding and non-responders patients (both mouse and human). This patient-oriented approach paves the way for the possibility of forecasting the best time for surgery or applying a watch-and-wait approach by a limited number of clinical observations during and early after NACRT, and in this paper, we discuss this perspective by analyzing available data in the literature on tumor volume measurements of patients with LARC that undergoes to standard preoperative treatment with NACRT [36,37]. We looked for data produced using magnetic resonance imaging (MRI) scans instead of computed tomography (CT) imaging for its precision in staging colorectal cancer of advanced stage [38].

## 2. Methods

The starting point is the observation that the Gompertz law (GL), initially applied to human mortality tables (i.e., aging), and the Logistic law (LL) describe tumor evolution. The GL and LL growth law can be written in general as (see Appendix A):

(1) $$\frac{1}{N(t)}\frac{dN(t)}{dt}=f\left[N\left(t\right)\right]$$

A constant value assumed by f[N(t)] gives exponential growth. In this case, a tumor progression starts from cells that collect genomic alterations. The produced malignant cells divide uncontrollably, and, in optimal conditions, one gets an exponential law. This pattern describes an uncontrolled tumor evolution but not that one in living organisms, where this expansion is “boundary driven” by multiple factors. For example, in a tumor spheroid, several cells in its center receive insufficient nutrients, creating a necrotic core. The outer part contains quiescent cells, which remain alive but lose their ability to replicate for nutrient deficiency, and the proliferative area, the outermost one, with free access to nutrients. More generally, it has been shown [39] that Gompertzian tumor growth can be reproduced by mitosis, related to nutrient supply, with local spatial cell correlations. The global metabolic energy supply constraint alone does not reproduce in vivo data according to the observed values of the nutrient expenditure for the cell activities. The depletion of the exponential growth, described by the Gompertz law, is obtained by nutrient supply conditions together with mean field spatial correlations or with a network formation among cells. It is clear, therefore, that growth in a realistic case cannot be exponential and is influenced by boundary conditions that control its maximum size. In particular, the GL and the LL depend on two parameters, which for cancer are related to the initial exponential trend and to the maximum number of cells, $N_{\infty }$, called carrying capacity, that can be supported by the local micro-environmental conditions (angiogenesis, immune system activity, nutrient supply, hypoxia, cell correlations, etc.). In a recent analysis [40], three classical models (exponential, logistic, and Gompertz) of tumor growth have been compared using a population approach. The Gompertz law, the solution of the differential equation,

(2) $$\frac{1}{N(t)}\frac{dN(t)}{dt}=k\;\ln\frac{N_{\infty }}{N(t)},\;$$

showed excellent descriptive power. For homogeneous systems, $N_{\infty }$ corresponds to the volume carrying capacity, $V_{\infty }$, and we consider such systems although the approach can be easily generalized to non-homogeneous ones. The carrying capacity (CC) changes according to some “external” conditions in many biological, economic, and social systems, and in tumor growth, its modification is related to a multi-stage evolution. In population dynamics, new technologies affect how resources are consumed, and since the carrying capacity depends on the availability of that resource, its value changes. Therefore a simple method of monitoring tumor evolution is to understand how the macroscopic parameters change for each specific patient during and after NACRT.

We carried out this analysis for colon-rectal cancer on the basis of the available data in the literature on tumor volume regression. Specifically, one dataset, pulled from the work of Bostel et al. [37], regards 8 patients with LARC that completed NACRT, addressing pathological complete response and pathological partial response to the treatment. Patients underwent therapy with 5-fluoruracil plus 50.4 Gy in 28 fractions, and GTV measurements were performed daily thanks to MRI used prior to irradiation (amounting to 171 datapoints, 76% of the total). In the second dataset, collected from the paper of Van den begin et al. [36], 15 patients were also treated with the standard preoperative NACRT with 50 Gy in 25 fractions and oral capecitabine. GTV was assessed before, during and after NACRT by eight MRI scans for each patient, totaling 120 measurements.

Notice that the proposed approach is dependent on the usual way of predicting the therapy effects, as the linear-quadratic model for radiotherapy, since it is based on the direct evaluation of the change of the macroscopic parameters during therapy. The final results show a clear, quantitative difference in the macroscopic evolution parameters among responders and non-responders patients. The dependence of the GL and LL on two parameters only suggests the possibility that a reliable, quantitative indication of the NACRT effects can be obtained by a small number of clinical observations of the tumor size regression during and after the treatment, without following the day-by-day evolution but monitoring it at regular interval. Therefore, the selection among partially responding patients (PR) and complete recovery (CR) cases should be possible much earlier than the standard time for surgery. The standard treatments for LARC patients require 5–6 weeks of NACRT followed by 6–8 weeks for TME. The total period of treatment can keep 11–14 weeks overall. Our approach can be helpful for an earlier clinical decision about the redefinition of the treatment, where possible, aimed at a watch-and-wait approach and/or an early or late surgery.

## 3. Results

The tumor size regression in colon-rectal cancer during and after NACRT has been experimentally studied in refs. [36,37] for 23 patients. The results in ref. [36], concerning the average gross tumor volume (GTV) reduction for 15 patients, and the data in ref. [37], for 8 individual (PR and CR) patients during *n* treatments in about 28 days, have been successfully compared with the GL and LL in ref. [31]. The fitted values of the CC, obtained by the whole data set and reported in Table 1, show a strong difference among PR and CR patients: the CC for CR cases is much smaller than the PR patient ones.

The difference in behavior between PR and CR patients is so strong that the question arises if the late response to the therapy can be predicted on the basis of a limited sequence of early treatments. If this conclusion is supported by data analysis, one could evaluate, with some uncertainty, the time evolution during and after the end of NACRT to obtain some useful information on the best time for surgery. On the other hand, the restriction to a subset of the whole time series requires a criterion to understand the number of data to be used for a reliable prediction. To clarify the adopted heuristic selection method, let us consider, for example, that for a specific patient, the GTV regression data, for n=23 NACRT treatments, have been collected during 28 days and that the CC fitted by the complete data series is 20 cm${}^{3}$. Let us now fix a subset of n<23 data to fit the GL evolution parameters. The data subset cannot be too small for two reasons. The first one is that after a small number of doses, *n*, the answer is obviously unreliable. The second motivation is more technical and depends on the result (see Appendix A) that the disentanglement between the GL (or the LL) and an exponential regression pattern, which has no finite CC, could require a long enough time series. This problem is more relevant for the complete recovery cases characterized by a very small CC. However, by increasing the number of data, *n*, to fit the CC, i.e., CC(*n*), its value will converge to that one obtained by the complete data set. Therefore, by increasing *n* and comparing CC(*n*) with CC(n+1), one should observe a convergent behavior with a small difference between CC(*n*) and CC(n+1) for large *n*. Let us, therefore, assume to take the value of *n* when the difference between CC(*n*) and CC(n+1) is less than about 10–15% for not very small *n* (to avoid the two unfortunate reasons mentioned above), and let us compare the CC obtained by this method (Table 2) with its value corresponding to the whole data set (Table 1). The application of this analysis to a subset of data in ref. [37] gives the result reported in Table 2 and the comparison with the CC of the complete dataset, in Table 1, implies that one can clearly differentiate among PR and CR using about 50% of the total number of treatments.

**Figure 1:**
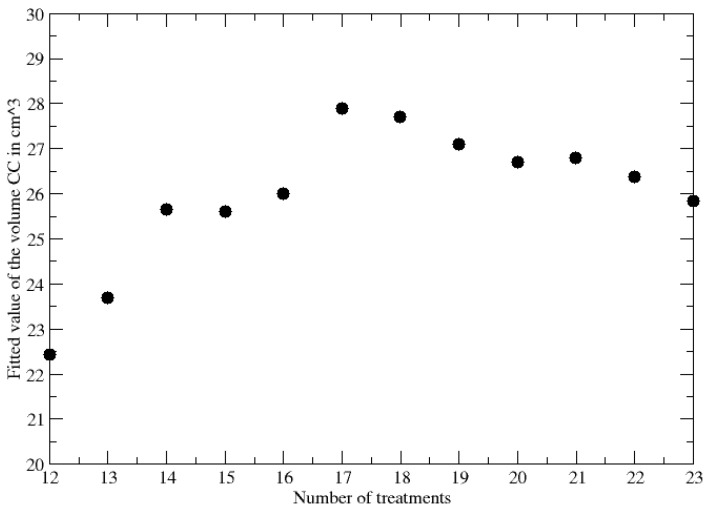
Convergence of the fitted CC by increasing the number of fitted data.

The results in Table 2 and Table 3 confirm that the approach gives clear information about the GTV for a later time, selecting among PR and CR patients. The better agreement for PR patients with respect to CR ones is related to the smallness of the final GTV in the latter cases. Finally, the convergence of the CC to its value obtained by the whole time series can be further verified by reporting its estimate as a function of the increasing data subset. The result for the patient PR1 is reported in Figure 1.

The suggested method can also be applied to determine the GTV regression after the end of NACRT. Indeed, an analogous study can be performed for the data in ref. [36] by using the CC fitted by data during NACRT to predict the evolution after the end of the treatment. The estimated GTV, 22 days after the end of NACRT, is about 35% of its initial value, and the experimental result is 38%. After 54 days one gets the predicted GTV ≃25% of the initial volume versus the observed 33%. Therefore, one deduces that the effect of waiting for 32 days reduces the GTV of 10% only. In this case, early surgery should be more useful. A further check concerns the reliability of the parameter values fitted by the specific data subset. This aspect can be tested by predicting the later time GTV regression by the CC in Table 2. For example, for patient PR1 in Table 2, the last used data is after 12 treatments, corresponding to 15 days from the beginning of the therapy, and by the corresponding CC, one estimates the GTV after the subsequent doses. This prediction is therefore compared with the observed GTV in Table 3, where the second column reports the day corresponding to the last dose included for the CC determination in Table 2. The other columns give the comparison between the observed data and the corresponding predictions. Notice that for patient CR4, one needs a large subset of data since the difference between the GL and an exponential behavior requires a long time series (see Appendix A).

According to previous results, the clear difference among PR and CR patients is encoded in the CC: for the CR cases, the corresponding CC are very small. Therefore the GL fit is similar to an exponential regression trend plus a constant factor and a simplified version of our hypothesis can be formulated by the comparison between an exponential fit with CC for PR and CR patients, i.e., by the equation:

(3) $$V\left(T\right)=V\left(0\right)*e^{\left[-k\left(t-t_{0}\right)\right]}+V\infty$$

with $V_{\infty }<<1$ cm${}^{3}$ for CR. Although the agreement with data of this approximated approach is worse than the corresponding GL (or LL) fit, the evaluated difference in the CC among PR and CR cases is still so large, allowing for an early reliable distinction between the two groups. In fact, the CC obtained by the whole data set fitted by Equation (3) are reported in Table 4.

According to our hypothesis, one has to verify if the result in Table 4 persists when a data subset is taken into account. In Table 5 is reported the fitted CC by Equation (3) and by the data subset considered in Table 2. The comparison with Table 4 confirms the clear difference among PR and CR patients by this simplified method, although with a worse agreement with data.

## 4. Discussion

The comparison between Table 1 and Table 2 strongly supports the possibility of a patient-oriented forecast of the GTV by the initial observation of its regression. The result is weakly dependent on the considered macroscopic evolution law. Indeed, similar conclusions can be drawn by the LL or by the elementary exponential trend plus CC in Equation (3). The preferential application of the GL originates from a better fit of the tumor growth available data [40]. Let us then discuss its application as a tool to evaluate the best time for surgery in colon-rectal cancer after NACRT. The usual NACRT protocol lasts about 33 to 38 days, with five daily doses per week, and the best time for surgery is considered between 8 and 12 weeks after its completion. The whole time interval is 12–16 weeks. Let us assume a weekly measurement of the GTV during NACRT. According to the previous analysis, the number of data for a reliable prediction is about 50% of the whole time series. Therefore with six measurements, i.e., about 42 days, with four observations during therapy and two measurements of GTV after the end of treatment, one verifies if the specific patient is partially responding to the therapy or whether a complete recovery is possible. The CC by GL or by the elementary exponential fit in Equation (3) can be evaluated using the initial set of data. Following this point of view, the PR patient will have a larger CC, and the CR patient’s data will be fitted by a very small CC. Accordingly, the later evolution can be predicted with some uncertainty related to data precision. More precisely, the suggested procedure is: (a) to estimate, by the fitted CC from the initial data, the GTV regression for a later time, that is 8–12 weeks after the end of therapy; (b) to compare this value with the last available observed GTV data. If the difference is, say, less than 20%, therefore the effect of waiting for more weeks turns out to be relatively small, and consequently, one could decide on early surgery. The other way around, if the fitted CC is small, a CR is expected and the surgery should be delayed or canceled according to the watch-and-wait approach.

## 5. Conclusions

The proposed criterion to forecast the best time for surgery is based on a data subset selection to obtain a fit of the evolution parameters. Therefore, it is more reliable if a larger number of data is available, which requires a careful detection of the tumor size with a specific time schedule. Additionally, although our computational approach has been validated by two different data sets, the predictive method should be tested by other devoted experiments and also in other scenarios, for example, for the early detection of those tumors requiring a stereotactic boost due to a likely not complete clinical response following an initial RT course [41,42]. Moreover, an application of this criterion could be helpful also in identifying CR cases for the novel therapeutic approach in LARC based on Total Neoadjuvant Therapy (TNT). On the other hand, by a rather elementary fit of the GTV evolution during and immediately after the end of the therapy, one has clinical and patient-oriented indications to define the best therapy (surgery, other therapies).

## Figures and Tables

**Table 1:** GL volume CC parameter for PR and CR patients including the whole data set.

Patient	CC in cm${}^{3}$
PR1	25.83
PR2	11.5
PR3	<25.0
PR4	24.45
CR1	<<1
CR2	<<1
CR3	<<1
CR4	<<1

**Table 2:** GL CC parameter for PR and CR patients obtained by a subset of time series data [37].

Patient	Used/Total Number	CC
PR1	12/23	22.42
PR2	9/12	21.7
PR3	9/16	25.64
PR4	8/22	26.94
CR1	12/25	<<1
CR2	7/15	<<1
CR3	7/16	<<1
CR4	17/19	<<1

**Table 3:** GTV in cm${}^{3}$ predicted with the GL parameters fitted by the data subset.

Patient	Day Last Dose	Pred. Day	Pred./Obs.	Pred. Day	Pred./Obs.
PR1	15	23	30.25/31.4	28	27.6/28.3
PR2	16	22	24.5/20.6	28	23.2/17.5
PR3	16	21	31.7/32.6	28	29.6/25.2
PR4	14	21	27.2/24.4	28	27.4/24.4
CR1	16	21	8.45/5.4	28	5.5/2.8
CR2	9	24	6.2/2	28	4.3/≃1
CR3	9	20	3.7/2.7	25	2.5/1.9
CR4	23	24	5.4/6.2	27	4.2/≃1

Note: “day last dose” indicates the last dose used in the CC fitting procedure; “prediction day” (pred. day) is the estimated day for surgery; “predicted/observed” (pred./obs.) represents the predicted and observed GTV, during the tumor late tumor progression.

**Table 4:** CC parameter for PR and CR patients obtained by the exponential law plus CC fit in Equation (3) of the whole data set.

Patient	CC in cm${}^{3}$
PR1	29.26
PR2	14.46
PR3	4.54
PR4	24.64
CR1	0.00014
CR2	0.00006
CR3	0.0183
CR4	0.062

**Table 5:** GTV in cm${}^{3}$ predicted by the exponential eq. plus CC (3) parameters fitted by the data subset.

Patient	Used Data	CC
PR1	12/23	30.6
PR2	8/12	6
PR3	9/16	27.54
PR4	8/22	27.25
CR1	12/25	0.0026
CR2	7/15	0.147
CR3	7/16	0.085
CR4	17/19	0.04

## Data Availability

All data analyzed during the current study are drawn from [36,37].

## Abbreviations

The following abbreviations are used in this manuscript:

AJCC	American Joint Committee on Cancer
CC	carrying capacity
cCR	Clinical Complete Response
CR	Complete recovery
DOAJ	Directory of open access journals
GL	Gompertz law
GTV	Gross Tumor Volume
LARC	Locally Advanced Rectal Cancer
LD	Linear dichroism
LL	Logistic law
LNY	lymph node yield
LR	Local Recurrence
MDPI	Multidisciplinary Digital Publishing Institute
MRI	Magnetic resonance imaging
NACRT	Neoadjuvant chemoradiotherapy
NACT	Neoadjuvant chemotherapy
NAT	Neoadjuvant Therapy
NCCN	National Comprehensive Cancer Network
NOM	Non-operative management
OS	Overall Survival
PR	Partially responding
QoL	Quality of Life
RT	Radiotherapy
TLA	Three letter acronym
TME	Total Mesorectal Excision
TNT	Total Neoadjuvant Therapy
TRG	Tumor Regression Grade

## Appendix A

The GL, for homegeneous systems, is the solution of the equation

(A1) $$\frac{1}{V}\frac{dV}{dt}=kln\left(\frac{V_{\infty }}{V}\right),$$

where $V_{\infty }$ is the volume CC. Therefore, the GTV evolves in time according to

(A2) $$\frac{V(t)}{V(t_{0})}=e^{ln\left(\frac{V_{\infty }}{V_{0}}\right)\left[1-exp\left(-k\left(t-t_{0}\right)\right)\right]}$$

where $V_{0}$ is the initial ($t_{0}$) value. It is useful to clarify that, in some cases, one needs a long series of observations to disentangle the GL behavior from an exponential one. Indeed, if $k(t-t_{0})<<1$, the solution can be approximated as

(A3) $$\frac{V(t)}{V(t_{0})}=e^{ln\left(\frac{V_{\infty }}{V_{0}}\right)*k\left(t-t_{0}\right)}$$

which is an exponential behavior. In such a case, the independent determination of the two GL parameters, $V_{\infty },k$ cannot be done unambiguously since the $\chi^{2}$ is extremely flat in a large parametric region and only the product $ln(\frac{V_{\infty }}{V_{0}})*k$ can be obtained by fitting the experimental data.

Concerning the LL, it is the solution to the logistic equation

(A4) $$\frac{1}{V(t)}\frac{dV}{dt}=\lambda \left(\frac{V_{\infty }}{V}-1\right).$$

and it is given by

(A5) $$V\left(t\right)=V_{\infty }\left[1+\left(\frac{V_{0}}{V_{\infty }}-1\right)*e^{\left(-\lambda \left(t-t_{0}\right)\right)}\right].$$
